# Differential upregulation of AU-rich element-containing mRNAs in COVID-19

**DOI:** 10.1186/s40246-022-00433-9

**Published:** 2022-11-15

**Authors:** Tala Bakheet, Khalid S. A. Khabar, Edward G. Hitti

**Affiliations:** Molecular BioMedicine Program, Research Centre, King Faisal Specialist Hospital and Research Centre, Riyadh, 11211 Saudi Arabia

**Keywords:** AU-rich elements, Post-transcriptional gene regulation, COVID-19

## Abstract

**Background:**

AU-rich elements (AREs) are located in the 3′UTRs of 22% of human mRNAs, including most transiently expressed inflammatory mediators. By default, AREs mark mRNAs for decay and translational inhibition, but this activity can be temporarily inhibited in case of infection to allow the onset of inflammation. Morbidity and mortality in COVID-19 patients have been associated with dysregulated inflammation, a process that may include aberrant ARE activity.

**Results:**

RNA-seq data from available transcriptomic studies were analyzed to investigate a possible differential expression of mRNAs that contain AREs in the context of SARS-CoV-2 infections. ARE-mRNAs turned out to be significantly overrepresented among the upregulated mRNAs after SARS-CoV-2 infection (up to 42%). In contrast, ARE-mRNAs were underrepresented (16%) in the downregulated group. Consequently, at a global scale, ARE-mRNAs are significantly more upregulated after SARS-CoV-2 infection compared to non-ARE mRNAs. This observation was apparent in lung cell line models such as A549 and Calu-3 and with infections with other respiratory viruses and cell lines. Most importantly, at the clinical level, the elevated ARE-mRNA response appeared strongest in blood cells of COVID-19 patients with mild disease. It diminished with disease severity and was least apparent in patients in need of intubation and respiratory-related death. Gene function and clustering analysis suggest that the ARE-response is rather global and the upregulated ARE-mRNAs in patients with mild disease do not particularly cluster in specific functional groups.

**Conclusions:**

Compared to the rest of the transcriptome, ARE-containing mRNAs are preferentially upregulated in response to viral infections at a global level. In the context of COVID-19, they are most upregulated in mild disease. Due to their large number, their levels measured by RNA-seq may provide a reliable indication of COVID-19 severity.

**Supplementary Information:**

The online version contains supplementary material available at 10.1186/s40246-022-00433-9.

## Background

Infection of human cells by viruses results in inflammation, a reaction that consists of concerted series of cellular and molecular responses that start the process of immune defense with the physiological purpose of restoring tissue homeostasis [1]. At the innate cellular level, pathogen-associated molecular patterns (PAMPs) activate cellular signaling events that induce the expression of inflammatory mediators like chemokines and cytokines. While necessary for the elimination of the threat, this response to pathogens such as the SARS-CoV-2 virus may become dysregulated leading to cytokine storm and death [2, 3].

The NF-kB pathway is a central element that is activated in response to pathogenic viral infections. Its activation results in the induction of the transcription of hundreds of pro-inflammatory genes to initiate and control inflammation [4, 5]. In parallel, interferon response is initiated which consists of a complex network of gene expression that initiates a cellular state of viral resistance within host cells [6].

The inflammatory response is not limited to transcription; equally important and vital is post-transcription. In the context of inflammation, the AU-rich elements (AREs)-dependent post-transcription is of particular importance [7–10]. AREs are located in the 3′UTRs of up to 22.4% of human mRNAs including most inflammatory cytokines, interferons, chemokines and transiently expressed genes [11–13]. They block expression during homeostasis and repress cytokine expression to terminate inflammation since they confer instability and inhibit translation of cis-mRNAs, reviewed in [14]. During the onset of inflammation, and when the expression of inflammatory cytokines is needed, the activity of AREs is temporarily inhibited by host cellular phosphorylation signaling events, typically the p38 MAPK signaling cascade. Dysregulated activity of this and other pathways contribute to disease conditions like chronic inflammation, immune disorders or cancer [15–17].

Several recent studies conducted RNA-seq-based transcriptomic studies to investigate SARS-CoV-2-dependent differential gene expression. Blanco-Melo et al. conducted such an approach to investigate the global transcriptomic response of cell lines and clinical samples to upper respiratory illness virus infections including SARS CoV-2. The authors concluded that compared to the other viruses, SARS-CoV-2 infection drives a lower antiviral interferon response but a higher cytokine response [18]. Other similar studies were conducted on clinical samples, with comparable conclusions [19–22].

Here, available RNA-seq transcriptomic data were analyzed to investigate a possible differential expression of ARE-containing mRNAs in the context of viral infections. It turned out that this group of genes as a whole is more likely to be upregulated in response to viral infection than mRNAs that lack AREs in their 3′UTR. Interestingly, marked differences were observed between SARS-CoV-2 and other viruses as well as in patients with mild COVID-19 compared to those with severe disease.

## Results

### A preferential ARE-mRNA upregulation in cell lines infected with SARS-CoV-2 and other respiratory viruses

**Fig. 1:**
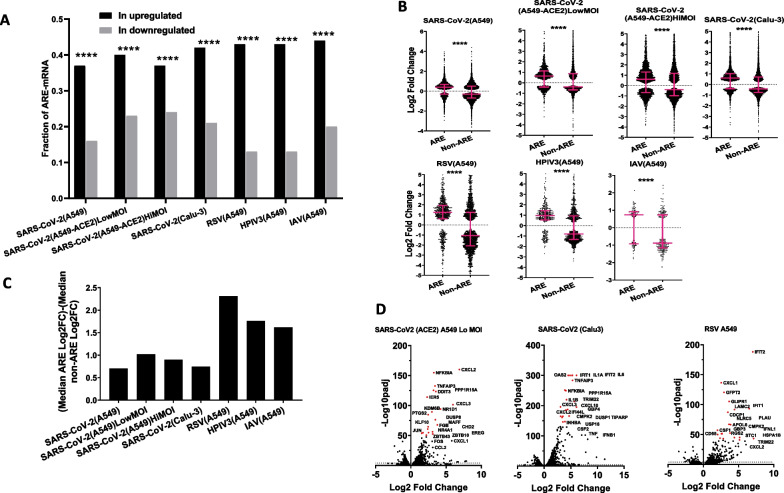
ARE-containing mRNAs are preferentially upregulated in virus-infected cell lines. **A** Fractions of ARE-containing mRNAs within the overexpressed and under-expressed groups of mRNAs in virus-infected cells. Virus and cell lines are indicated, ACE2 depicts cells expressing the receptor. MOI (Multiplicity of infection). *P* value for difference in population proportion depicted as ****(*P* < 0.0001). **B** Log2 Fold Change of uninfected control of ARE- and non-ARE mRNA levels are displayed. Significantly differentially expressed mRNA (Padj < 0.05) were split into those that contain AREs and those that do not (non-ARE) and displayed as a dot plot. Medians with interquartile ranges are displayed. The types of cell lines and infecting viruses are depicted. Statistical significance was determined by Mann–Whitney t test in Graph prism and *P* value displayed in graph ****(*P* < 0.0001). **C** The difference between medians of the Log2 Fold Change of ARE-mRNA and non-ARE mRNA in the indicated infected cell lines are displayed. **D** Volcano plots, Log2 Fold Change against -Log10 of adjusted *P* value of differentially expressed ARE-mRNAs in selected types of cells lines and infecting viruses. The dots of the 20 most significantly differentially expressed mRNAs are colored in red and their names are depicted

Several recent studies used RNA-seq-based global transcriptomic analysis to investigate differential gene expression resulting from SARS-CoV-2 and other viral infections. In such a study, the human lung cancer cell line A549 was used as a model to assess the global effect on gene regulation of the following viruses: SARS-CoV-2, respiratory syncytial virus (RSV), human parainfluenza virus type 3 (HPIV3) or influenza A virus (IAV). In the same study, another lung cancer cell line Calu-3 was also infected with SARS-CoV-2 [18]. The authors concluded that compared to other viral infections, host response to SARS-CoV-2 is deficient in interferon response, but is excessive in inflammatory cytokine and chemokine production [18]. Here, we determined the fraction of ARE-containing mRNAs within the groups of infection-dependent upregulated or downregulated genes by crossing the list of differentially expressed genes (DEGs) with the AU-rich element (ARED-plus) database [12]. The statistical significance for differential expression was set to adjusted *P* value < 0.05. Interestingly, in all cases where cell lines were used, the fraction of ARE-containing mRNAs within the overexpressed genes was higher than the ARE-fraction in the downregulated mRNAs with very high statistical significance (Fig. 1A; Additional file 1: Table S1: Upregulation). For instance, 37% of the upregulated mRNAs in SARS-CoV-2-infected A549 cells contained AREs and only 16% of the downregulated. Similar results were observed in A549 expressing the SARS-CoV-2 receptor protein ACE2 with high multiplicity of SARS-CoV-2 infection (MOI) (37 vs. 24%) or low MOI (40 vs. 24%). A similar observation was also made in Calu-3 cells infected with SARS-CoV-2 (42 vs. 21%). Other viruses transfecting A549 cells like RSV or HPIV3 or IAV had similar but more pronounced effects. Most notably was RSV (43 and 13%). The median of the Log2 Fold Change between transfected versus un-transfected was always clearly and significantly higher in ARE-mRNAs compared to the non-ARE mRNAs. Due to the large sample sizes of ARE and non-ARE mRNAs, the results were always very highly significant with very low *P* values typically less than 10^–17^ and consequently with a narrow range of 95% CI (data not shown) and low margin of error.

Importantly, the difference between the median of Log2 Fold Change of ARE and that of the non-ARE was higher in A549 infected with RSV, HPIV3, IAV compared to the same cell line and Calu-3 infected with the SARS-CoV2, suggesting that SARS-CoV-2 drives a weaker ARE-dependent response than the other viruses (Fig. 1B, C).

Volcano plots of ARE-mRNAs in SARS-CoV-2-infected A549 and Calu-3 and RSV-infected A549 are displayed and show that all of the 20 most significantly differentially expressed ARE-mRNAs are overexpressed and none is under-expressed (Fig. 1D). Similar observations were made in all other infections (data not shown). Among the recurring, most significantly upregulated ARE-mRNAs are typically cytokines and chemokines like CXCL1, CXCL2, TNF and IL6 but also regulatory genes like TNFAIP3, NFKBIA, DUSPs and JUN (Fig. 1D).

**Fig. 2:**
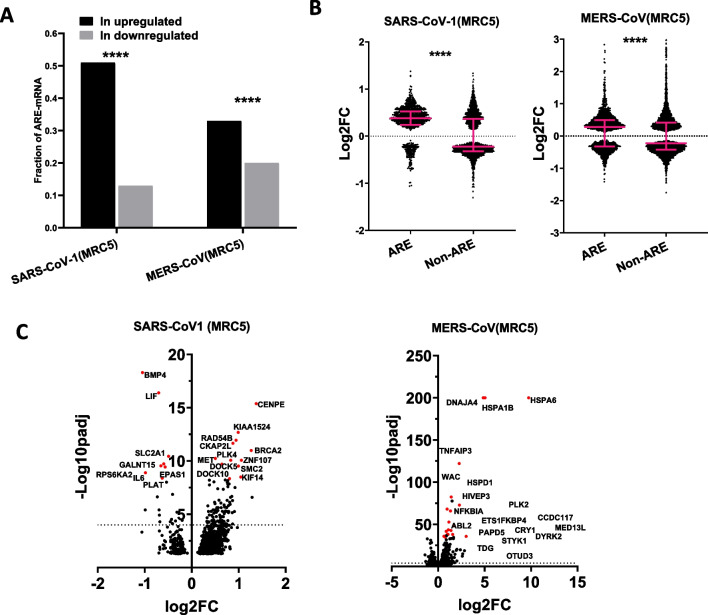
ARE-containing mRNAs are preferentially upregulated in virus-infected MRC5 cell lines. **A** Fractions of ARE-containing mRNAs within the overexpressed and under-expressed groups of mRNAs in SARS-CoV-1- and MERC-CoV-infected MRC5 lung cells. Virus and cell lines are indicated. *P* value for difference in population proportion depicted as ****(*P* < 0.0001). **B** Log2 Fold Change of ARE- and non-ARE mRNA levels are displayed. Significantly differentially expressed mRNA (Padj < 0.05) were split into those that contain AREs and those that do not (non-ARE) and displayed as a dot plot with medians with interquartile ranges. The types of infecting viruses are depicted. Statistical significance was determined by Mann–Whitney *t* test in Graph prism and *P* value displayed in graph. **C** Volcano plots, Log 2 Fold of control against Log10 of adjusted *P* value of overexpressed ARE-mRNAs in selected different types of cells lines and infecting viruses. The dots of 20 most significantly differentially expressed mRNAs are colored in red and their names are depicted

Similarly, the transcriptomic effects of SARS-CoV-1 and MERS-CoV infection of the human fetal lung fibroblast cells MRC5 were analyzed (GSE56192) and [18]. A notable 51% of overexpressed mRNAs in the SARS-CoV-1-infected MRC5 cells contained AREs, whereas only 13% of the downregulated (Fig. 2A). A significant but less pronounced effect on MRC5 was observed in MERS-CoV (33 and 20%) (Fig. 2B). Volcano plots show that all or most of the 20 most significantly differentially expressed mRNAs in MERS-CoV-1 and SARS-CoV-1 are ARE-containing (Fig. 2C). Taken together, these in vitro observations clearly state that cell lines infected with respiratory viruses tend to overexpress ARE-containing mRNA more than non-ARE, and this observation is weaker with SARS-CoV-2 compared to other viruses.

### ARE-mRNA levels in SARS-CoV-2-infected clinical samples

**Fig. 3:**
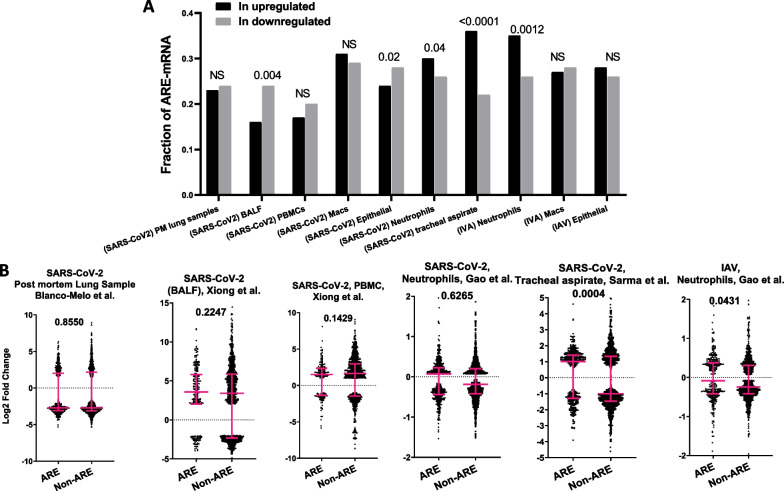
ARE- and non-ARE-containing mRNAs levels in virus-infected clinical samples. **A** Fractions of ARE-containing mRNAs within the overexpressed and under-expressed groups of mRNAs in virus-infected clinical samples. Data in this figure originate from four sources [18–20, 22]. Virus and type of clinical sample depicted. PM = post-mortem, BALF = bronchoalveolar lavage fluid. *P* value for difference in population proportion depicted as ****(*P* < 0.0001). **B** Log2 Fold Change of ARE- and non-ARE mRNA levels in infected versus healthy samples are displayed. Significantly differentially expressed mRNA (Padj < 0.05) were split into those that contain AREs and those that do not (non-ARE) and displayed as a dot plot. Medians with interquartile ranges are displayed. Statistical significance was determined by Mann–Whitney *t* test in Graph prism and *P* value displayed in graph

**Fig. 4:**
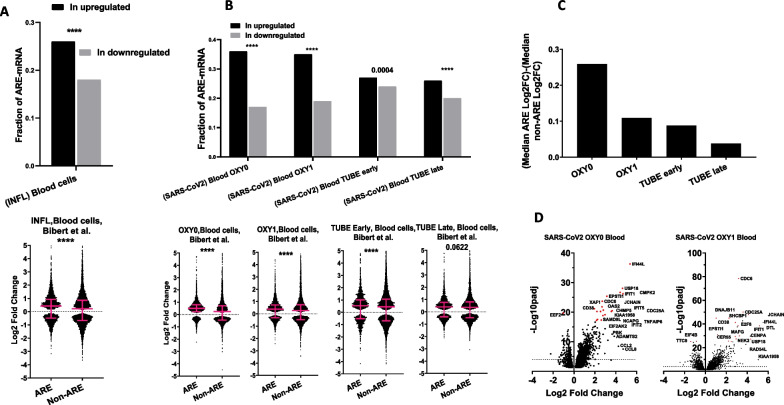
ARE-containing mRNAs are especially upregulated in blood cells of SARS-CoV-2 patients with influenza and mild COVID-19 symptoms. **A** upper panel, fractions of ARE-containing mRNAs within the overexpressed and under-expressed groups of mRNAs in blood cells of influenza A and B infected clinical samples. Significance of the difference in population proportion depicted as ****(*P* < 0.0001). Lower panel, Log2 Fold Change of ARE- and non-ARE mRNA levels in infected versus healthy samples are displayed. Significantly differentially expressed mRNA (Padj < 0.05) were split into those that contain AREs and those that do not (non-ARE) and displayed as a dot plot. Medians with interquartile ranges are displayed. Statistical significance was determined by Mann–Whitney t test in Graph prism and *P* value displayed in graph ****(*P* < 0.0001). **B** Same as A but with samples with different severity levels of COVID-19: Upper panel, fractions of ARE-containing mRNAs within the overexpressed and under-expressed groups of mRNAs in blood cells of patients with four different levels of symptom severity; from least severe OXY0 to most severe TUBE late. Lower panel, Log2 Fold Change of ARE- and non-ARE mRNA levels in infected versus healthy samples is displayed in a dot plot. **C** The difference between medians of the Log2 Fold Change of ARE-mRNA and non-ARE mRNA in the indicated COVID-19 severity levels are displayed **D** Volcano plots, Log2 Fold Change against Log10 of adjusted *P* value of the differentially expressed ARE-mRNAs in the mild COVID19 samples OXY0 and OXY1. The names of most significantly differentially expressed mRNA are depicted

Similar to the investigations performed on cell lines above, a possible higher upregulation of the ARE-mRNA containing fraction from clinical samples was investigated. First, we analyzed transcriptomic data originating from post-mortem lung samples [18]. SARS-CoV-2 infection did not have a significant enrichment in the ARE-gene fraction within the overexpressed group of mRNAs and subsequently no higher global levels of ARE-mRNAs could be detected (Fig. 3A, B). Xiong et al. [19] investigated the differential expression of mRNAs in bronchoalveolar lavage fluid (BLAF) and PBMCs of SARS-CoV2-infected patients. Our analysis shows no significant enrichment of the ARE-mRNA fraction in the upregulated group; in fact, a tendency to the opposite, especially in BALF (16 vs. 24%), could be observed; however, this did not result in a significantly higher global upregulation of ARE-mRNAs (Fig. 3A, B). Analysis of transcriptomic studies on neutrophils, epithelial cells and macrophages from infected individuals compared to healthy donors resulted in a comparatively weak preferential enrichment and upregulation of ARE-mRNAs in neutrophils only. This, however, did not result in a global preferential upregulation of ARE-mRNAs [20]. In the same study, the authors investigated the effect of IVA infection in the same type of cells. The enrichment of the ARE-signature within the overexpressed genes was observed and is significant only in neutrophils infected with IAV (35 vs. 26%), and this resulted in a comparatively weak enrichment and upregulation of ARE-mRNAs (Fig. 3A, B). Sarma et al. [22] performed transcriptomic studies on tracheal aspirate from COVID-19 patients with acute respiratory distress syndrome (ARDS) with uninfected controls without ARDS. We found a significant enrichment of the ARE-mRNAs within the overexpressed genes (36 vs. 22%) along with a higher upregulation of ARE-mRNAs compared with those that do not contain AREs (*P* = 0.0004) (Fig. 4A, B).

Overall, the results of the investigation of clinical samples may suggest that the higher upregulation of ARE-mRNAs observed in SARS-CoV-2-infected cell lines is non-existent or weak in clinical samples compared to cell lines. However, it is important to note that the Blanco Mello et al. and Xiong et al. transcriptomic data were based on only two and three SARS-CoV-2 patients and healthy donors and are likely to be lacking the statistical power to identify transcripts with moderate but significant differential expression. Similarity, the Gao et al. study was based on five SARS-CoV-2 and IAV samples. The transcriptomic data from Sarma et al. however, was based on 15 COVID-19 patients and 5 controls and resulted in a significant ARE-mRNA upregulation signature.

### Preferential ARE-mRNA upregulation in blood cells of patients with mild forms of COVID-19

Bibert et al. [21] conducted a large comparative transcriptomic profile study that is based on blood cells from 103 patients with different severity levels of COVID-19 along with 27 healthy and 22 influenza-infected individuals. The authors classified COVID-19 patients into four groups according to the level of respiratory failure; 23 with no oxygen support requirements (OXY0), 40 who received oxygen but no need for mechanical ventilation (OXY1), 15 who required mechanical ventilation within the first 7 days in hospital (TUBE early) while 25 were sampled after 7 days (TUBE late). Log2 Fold Change for influenza infection and each of the four severity groups was determined using DESeq2 at a transcriptomic level compared to the healthy donors. The influenza A and B virus-infected patients displayed the typical ARE-over-representation and expression signature found in cell lines (Fig. 4A). Most interesting was the OXY0 group of SARS-CoV-2-infected individuals: a strong and highly significant enrichment of ARE-mRNAs within the upregulated mRNAs of infected patients compared to the downregulated group could be observed (36 vs. 17%). This correlated with a clear and significant higher upregulation of ARE-mRNAs compared to non-ARE, median of Log2 Fold Change of ARE-mRNAs 0.5 versus 0.241 for non-ARE. (Fig. 4B, C). With the large sample sizes of ARE and non-ARE mRNAs, the result was very highly significant (*P* value = 9.26 × 10^–30^) and the 95% CIs were narrow (0.48–0.52 for ARE-mRNAs and 0.185–0.28 for non-ARE). Strikingly, this type of signature became reduced with increasing severity of the disease (Fig. 4B, C). For instance, for OXY1, the enrichment was 35% versus 19% and the medians 0.38 versus 0.27, while for TUBE early, the enrichment was comparatively weakly significant, 27 versus 24% (*P* = 0.0004), and the medians, 0.53 versus 0.44. For TUBE late, the enrichment was 26 versus 20%, and the difference in the Log2 Fold Change between ARE and non-ARE mRNAs was 0.4058 versus 0.3676 and no longer significant (*P* = 0.0622) (Fig. 4B, C). Similar to results from cells line, a volcano plot from OXY0 and OXY1 patients shows that most of the 20 most significantly differentially expressed ARE-mRNAs are overexpressed (Fig. 4D).

### ARE-mRNAs that are upregulated in the mild form of the disease

**Fig. 5:**
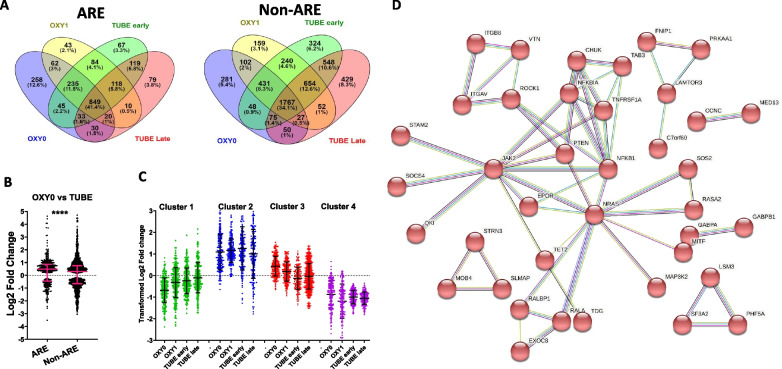
Analysis and functional clustering of upregulated ARE-mRNAs that are preferentially upregulated in the mild forms of COVID-19. **A** Left panel, Venn diagram of ARE-mRNAs that are upregulated in in blood sample of patients with four levels of disease severity OXY0, OXY1, TUBE early and TUBE late. Left panel, same as right panel but with mRNAs that lack AREs. **B** Log2 Fold Change of ARE and non-ARE mRNA levels in patients with no need of oxygen (OXY0) versus intubated patients (TUBE). Significantly differentially expressed mRNA (Padj < 0.05) were split into those that contain AREs and those that do not (non-ARE) and displayed as a dot plot. Medians with interquartile ranges are displayed and *P* value displayed in graph ****(*P* < 0.0001). **C** K-means clustering of the Log2 Fold Change values of the 849 mRNAs that were upregulated in OXY0, OXY1, TUBE early and TUBE late. The values were Johnson transformed with column scaling and clustered in JMP. **D** Cluster 3 from B was subjected to functional clustering in string and resulted in a functional cluster of 81 genes with highest significance

Since patients with mild disease have a higher overall signature of upregulated ARE-mRNAs, a Venn diagram was plotted to find ARE-mRNAs that are unique to patients with mild disease. For comparison, another diagram was plotted for non-ARE mRNAs (Fig. 5A). The largest proportion of upregulated ARE and non-ARE mRNAs was common to OXY0, OXY1, TUBE early and TUBE late, 41.3 and 34.1%, respectively. This suggests the existence of common regulatory networks that are activated in all levels of case severity of SARS-CoV-2. Interestingly, however, is a relative large proportion of upregulated ARE-mRNAs that are unique to the mildest form of COVID-19, OXY0, 12.6% (258 mRNAs) versus 5.4% (281 mRNAs) for non-ARE (Fig. 5A). Also interestingly and in contrast, the fractions of upregulated non-ARE mRNAs that are unique to TUBE early and TUBE late are twice the fractions in ARE-mRNAs. Precisely, 6.2 + 8.3 + 10.6 = 25.1% for non-ARE and 3.3 + 3.8 + 5.8 = 12.9% for ARE. These observations show that some upregulated ARE-mRNAs tend to be unique to the mild forms of the disease while some upregulated non-ARE mRNAs tend to be unique to the severe forms of the disease.

### Gene ontology analysis of ARE-mRNAs that are differentially up-regulated in mild COVID-19

The DESeq2 software was used to find mRNAs that are upregulated in the mildest form of the disease (OXY0) relative to patients in need of intubation (TUBE early and late). The median of the levels of ARE-mRNA in OXY0 relative to TUBE was higher than that of non-ARE mRNAs confirming a better ARE-response in the mild form of the disease (Fig. 5B). Next, the most significantly upregulated ARE-mRNAs in OXY0/TUBE (padj < 0.0001, *n* = 56) were subjected to gene ontology clustering in STRING portal (string-db.org). 27 (48%) turned out to be related to defense against viral infections and immune responses under the biological processes category (Additional file 2: Table S2). However, and similarly, 66 of 156 (42%) upregulated non-ARE mRNAs also clustered in same and similar categories that relate to defense against viral infections (Additional file 3: Table S3). The above observations indicate that the significant ARE-response is not alone responsible for the stronger interferon and immune response found in the mildest form of the disease.

Next, the 258 unique ARE-mRNAs that were upregulated in OXY0 (Fig. 5A) were analyzed using the STRING portal (string-db.org/). In the biological processes category, the highest strength of enrichment was weak, 0.77 in nucleotide excision repair (nine proteins of 111 of network, FDR 0.0159). All other enrichments in categories like molecular function, biological process, KEGG pathway and cellular component resulted in strengths of less than 0.5 indicating that the unique genes do not particularly cluster in common functional categories. Next, we focused on the 849 mRNAs that are upregulated in all four severity levels of COVID-19. Specifically, we searched for a possible cluster of ARE-mRNAs that has a higher upregulation level in the mild forms of the disease compared to the severe forms. The Log2 Fold Change values of all four forms were subjected to a K-means clustering in JMP resulting in four cluster. Cluster (3) consisted of 233 genes that are upregulated in OXY0 and OXY1 compared to TUBE early and TUBE late (Fig. 5C). The 233 genes clustered into three clusters using K-means in the string portal. Cluster 1 consisted of 81 genes and showed the highest enrichment (*p* value < 1.0e–16). PTEN signaling was central in this cluster and is in agreement with a previous observation [22] (Fig. 5D). KEGG pathway enrichments of the 81 genes were ranked according to strength and start with mTOR signaling but also contain other pathways including the anti-inflammatory PD-L1/PD-1 checkpoint pathway, viral infection pathways like human cytomegalovirus infection, human papillomavirus infection and chemokine signaling pathway (Additional file 4: Table S4).

## Discussion

AU-rich elements (AREs) reduce the stability and translation of many inducible mRNAs and are capable of responding to inflammatory cellular signaling. In this study, we propose that they can provide an informative integrated view on mRNA differential expression in response to viral infections. Several cellular signaling pathways induce ARE-containing mRNAs at the transcriptional level such as NF-kB, AP1, interferon, IL-6/JAK/STAT, MAPK kinases, Hippo, PI3K and others [23–26]. The same mRNAs can be regulated at the post-transcriptional level by the same and/or additional signaling pathways like p38 MAPK, PI3K, ERK, PLK1, PKC and other [14, 27, 28]. AREs are not involved in the regulation of transcription, but they are located in most mRNAs that are induced during inflammation at the transcriptional level. Therefore, they could be considered most downstream markers that integrate the effects of multitude of signaling pathways in inflammation; pathways that regulate both transcription and post-transcription. This integration can provide a view at a transcriptomic scale, i.e., the whole group of hundreds of ARE-mRNAs, whose levels are expected to be modulated by the mentioned signaling pathways that are triggered by an infection.

The identification of human ARE-containing genes was based on our latest database (ARED-Plus) [12]. To compile this database, we developed an algorithm to screen for AREs in the 3′UTR and introns of human mRNAs and pre-mRNAs. The algorithm relies on the presence of one or more overlapping AUUUA pentamers in a context of a region that is rich in U’s and As. It was optimized for maximum inclusion and comprehension, identifying 22.4% of mature transcripts as putative ARE-mRNAs. To a degree of certainty, the current analysis can be considered a functional biological validation of the algorithm used to compile the database.

The analysis of the ARE-signature response to SARS-CoV-2 in clinical samples was more challenging and interesting than the observations made in cell line models. As mentioned in the results section, some clinical studies relied on few, less than five samples of healthy and infected patients. This will likely allow only the detection of highly induced mRNAs due to lack of statistical power. In previous studies, we measured the “isolated” ARE-dependent post-transcriptional response to inflammation, using post-transcriptional reporter assays and it turned out to be in the range of only 1.5- to twofold of control much less than upregulation due to transcription of the combination of transcription and post-transcription which can be in the hundreds of folds [29–31]. Accordingly, this may be the reason for a lack of global ARE-upregulation signature in some clinical data originating from three of the investigated studies [18–20]. Therefore, and also due to the fact that clinical samples are heterogeneous compared to cell lines, a higher number of samples and higher statistical power should be necessary to detect a weak transcriptional or a post-transcriptional effect. Indeed, a highly significant ARE-upregulation signature could be observed when transcriptomic profiles of 103 COVID-19 patients of different disease severities were compared to 27 healthy donors [21]. Most importantly, the ARE-signature analysis of transcriptomic data originating from this study allowed the differentiation between patients with mild COVID-19 disease and those with severe disease. It turned out that patients with mild disease that do not need intubation (OXY0) have the strongest global ARE upregulation signature. This observation gradually decreased with patients that needed oxygen but no need for mechanical ventilation (OXY1), followed by patients that required mechanical ventilation within the first days in hospital (TUBE early), followed by patients that needed ventilation longer than seven days (TUBE late). These observations suggest that a high global ARE-upregulation response may contribute to mild disease.

At first, this interpretation may be considered unexpected since several inflammatory diseases have been linked to excesses in the expression of ARE-mRNAs [8]. This fact does not exclude the need for a well-regulated ARE-dependent post-transcriptional response as a natural and necessary process for healthy immunity; Only dysregulation leads to disease. Gene ontology analysis revealed some functional clustering of the favorably upregulated ARE-mRNAs in the mild clinical samples, like response to viral infections and signaling around PTEN. However, the response appears to be rather global involving most ARE-mRNAs and can provide an integrated birds eye view for the assessment of disease severity.

## Conclusions

This transcriptomic analysis was based on RNA-seq data from fully independent investigational models and resulted in a common conclusion: ARE-containing mRNAs have a higher tendency, as a group, to be overexpressed after viral infections than genes that do not contain AREs. In cell lines, the preferential upregulation of ARE-genes was significant and general to all investigated viruses including SARS-CoV1, SARS-CoV2, RSV, IAV, MERS-CoV and HPIV3. In clinical models, the effect was especially apparent when the analysis was based on large datasets. Most importantly, in COVID-19, the ARE-mRNA response was strongest in mild disease and decreased with disease severity. ARE-mRNAs make up to 22% the human transcriptome (> 4500 mRNAs), and they were compared to the remaining transcriptome (> 17,000 mRNAs). The large sample size of their levels may provide a reliable indication that facilitates the assessment of COVID-19 severity.

## Materials and methods

### RNA-seq transcriptomic data and AU-rich element mRNAs

NCBI’s PubMed and Gene Expression Omnibus (GEO) were extensively searched to find RNA-seq-based differential gene expression analysis resulting from SAR-CoV-2 and other viral infections. Transcriptomic data from A549 and/or Calu-3 cells transfected with SARS-CoV-2, RSV, HPIV3 and IAV were obtained from [18]. For this study, Illumina NextSeq 500 was used to generate sequencing libraries and RNA-Seq Aligment App to align raw reads to the human genome (hg19) using the on Basespace (Illumina, CA). We relied on author provided DEGs, however, in some cases adjusted P values (padj) were ambiguous; therefore, the DESeq2 software in R was used to compute the Log2 Fold Change and significance from deposited, assembled RNA-seq data (GSE147507). Our analysis was in agreement with author provided DEGs [18]. Transcriptomic data from MRC5 cells infected with SARS-CoV-1 and MERS-CoV originated from GSE56192 and were analyzed in [18]. Data originating from clinical patient samples were obtained from human tissues or cells type from several studies, relying on author provided DEGs. For instance, post-mortem lung samples from Blanco-Melo et al. [18]. Bronchoalveolar lavage fluid (BLAF) and PBMCs of SARS-CoV2-infected patients from Xiong et al. [19]. Macrophages, Epithelial cells and neutrophils from Gao et al. [20]. Tracheal aspirate from COVID-19 patients from Sarma et al. [22]. Extensive transcriptomic blood sample data from 103 COVID-19 patients, 22 influenza-infected patients and 27 healthy donors were from Bibert et al. [21]. The patients were classified into four groups depending on the level of respiratory failure: OXY0 (*n* = 23) with no need of oxygen, OXY1 (*n* = 40) received only oxygen, TUBE early (*n* = 15) required mechanical ventilation within the first 7 days in hospital and TUBE late (*n* = 25) required mechanical ventilation also after 7 days. Symptoms duration among the four severity groups was comparable and the baseline characteristics and comorbidities of patients were similar among all COVID-19 patients, there was an over-representation of males among the TUBE late group [21]. The study used Illumina HiSeq 4000 sequencer with reads of 100 bases and computed transcript abundance quantification on Salmon v1.3.0. A comprehensive search engine is available to find individual DEGs in blood of COVID-19 patients (https://bix.unil.ch/covid/). We used the provided assembled data to compute the Log2 Fold Change and padj using DESeq2 in R. DEGs from our analysis were compared with the author search engine by random sampling and were always in agreement. Log2 Fold Change with padj or FDR < 0.05 was set for statistical significance. The ARED-plus AU-rich element database was used to select ARE genes from the transcriptomic studies [12].

### Statistical analysis

To assess the significance between the ARE fractions in the downregulated and upregulated mRNAs in virus-infected cells, the *Z* value for difference in population proportion significance was calculated using the following formula: *Z* = (*p̂*_1_ − *p̂*_2_)/SQRT(p̄*(1 − *p̄*)/*n*_1_) + (p̄*(1 − *p̄*)/*n*_2_), where *p̂*_1_ = fraction of ARE-genes in downregulated and *p̂*_2_ = fraction of ARE genes in up-regulated and *p̄* = (*x*_1_ + *x*_2)_/(*n*_1_ + *n*_2_) and *x*_1_ = number of downregulated ARE genes and *x*_2_ = number of upregulated ARE-genes and *n*_1_ = total number of downregulated genes and *n*_2_ = total number of upregulated genes. *P* value was computed in Excel using NORM.S.DIST function (2*NORM.S.DIST) for two tailed. Graph prism was used for data plotting and statistical analysis of the Log2 Fold Change of the ARE versus non-ARE mRNAs in virus-infected cells or clinical samples. Medians with interquartile ranges were displayed. The nonparametric Mann–Whitney *t* test was used to assess statistical significance.

### Data display and clustering

The Venny portal was used to display the Venn diagrams (bioinfogp.cnb.csic.es/tools/venny/) and to investigate common genes within different groups. The Log2 Fold Change of the genes that were common to OXY0, OXY1, tube early and tube late was subjected to K-means normal mixture clustering in JMP^®^, Version <12.0.1> . SAS Institute Inc., Cary, NC, 1989–2021. The data were transformed by Johnson and columns were scaled individually. The number of clusters was set between 3 and 5. The cluster group with the smallest Bayesian information criterion (BIC) was selected for further analysis. The string portal (string-db.org/) was used for gene ontology.

## Supplementary Information

**Additional file 1:** Table S1.

**Additional file 2:** Table S2.

**Additional file 3:** Table S3.

**Additional file 4:** Table S4.

## Data Availability

The analyzed data during the current study are available from the corresponding author on reasonable request**.**
